# Supplementary material for: FAM134B isoform 2/RETREG1-2 defines a calnexin–TOLLIP–coupled ER-phagy pathway that restricts Ebola virus glycoprotein and is antagonized by VP40 through macro-autophagy
Source: bioRxiv. 2026 Apr 2:2026.04.01.715898. Preprint. [Version 1] doi: 10.64898/2026.04.01.715898 (PMC13054541; doi:10.64898/2026.04.01.715898)
Supplement: 1 [file NIHPP2026.04.01.715898v1-supplement-1.pdf]

bioRxiv preprint doi: https://doi.org/10.64898/2026.04.01.715898; this version posted April 2, 2026. The copyright holder for this preprint (which was not certified by peer review) is the author/funder, who has granted bioRxiv a license to display the preprint in perpetuity. It is made available under aCC-BY 4.0 International license.

A

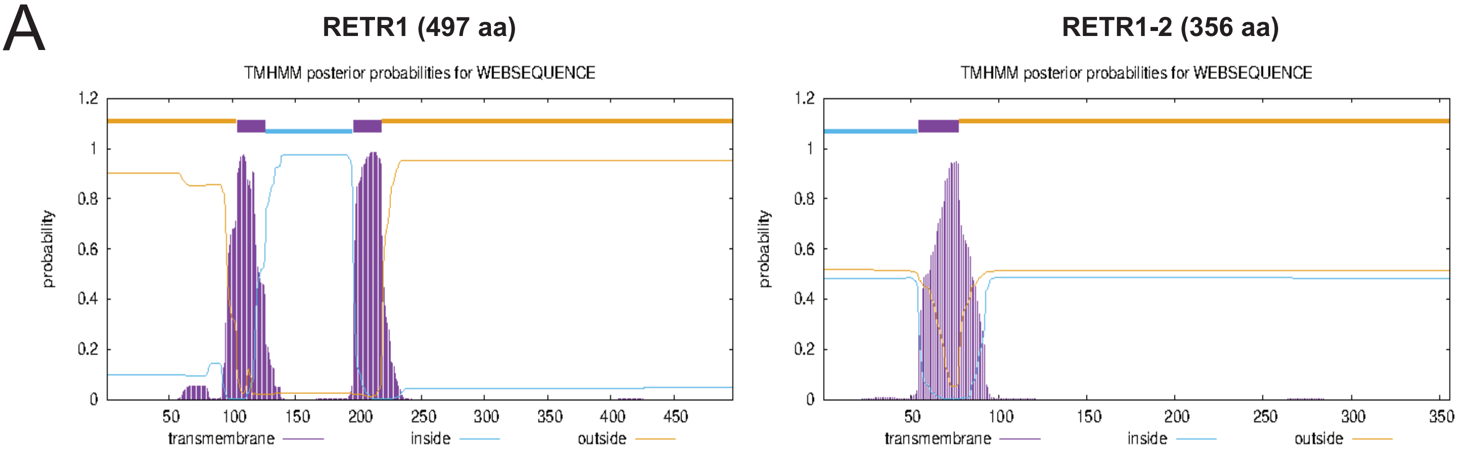

B

```
             1                                                                60
RETR1        MASPAPPEHA EEGCPAPAAE EQAPPSPPPP QASPAERQQQ EEEAQEAGAA EGAGLQVEEA
RETR1-2      ---------- ---------- ---------- ---------- ---------- ----------
             61                                                   TM1    120
RETR1        AGRAAAAVTW LLGEPVLWLG CRADELLSWK RPLRSLLGFV AANLLFWFLA LTPWRVYHLI
RETR1-2      ---------- ---------- ---------- ---------- ---------- ----------
             121                                    EBOV-GP recognition?180
RETR1        SVMILGRVIM QIIKDMVLSR TRGAQLWRSL SESWEVINSK PDERPRLSHC IAESWMNFSI
RETR1-2      ---------- ---------M PEGEDF--GP GKSWEVINSK PDERPRLSHC IAESWMNFSI
             181                     TM2                                 240
RETR1        FLQEMSLFKQ QSPGKFCLLV CSVCTFFTIL GSYIPGVILS YLLLLCAFLC PLFKCNDIGQ
RETR1-2      FLQEMSLFKQ QSPGKFCLLV CSVCTFFTIL GSYIPGVILS YLLLLCAFLC PLFKCNDIGQ
             241                                                         300
RETR1        KIYSKIKSVL LKLDFGIGEY INQKKRERSE ADKEKSHKDD SELDFSALCP KISLTVAAKE
RETR1-2      KIYSKIKSVL LKLDFGIGEY INQKKRERSE ADKEKSHKDD SELDFSALCP KISLTVAAKE
             301                                                         360
RETR1        LSVSDTDVSE VSWTDNGTFN LSEGYTPQTD TSDDLDRPSE EVFSRDLSDF PSLENGMGTN
RETR1-2      LSVSDTDVSE VSWTDNGTFN LSEGYTPQTD TSDDLDRPSE EVFSRDLSDF PSLENGMGTN
             361                                                         420
RETR1        DEDELSLGLP TELKRKKEQL DSGHRPSKET QSAAGLTLPL NSDQTFHLMS NLAGDVITAA
RETR1-2      DEDELSLGLP TELKRKKEQL DSGHRPSKET QSAAGLTLPL NSDQTFHLMS NLAGDVITAA
             421                                                         480
RETR1        VTAAIKDQLE GVQQALSQAA PIPEEDTDTE EGDDFELLDQ SELDQIESEL GLTQDQEAEA
RETR1-2      VTAAIKDQLE GVQQALSQAA PIPEEDTDTE EGDDFELLDQ SELDQIESEL GLTQDQEAEA
             481              497
RETR1        QQNKKSSGFL SNLLGGH
RETR1-2      QQNKKSSGFL SNLLGGH
```

Figure S1. (A) The transmembrane helices of RETR1 and RETR1-2 were precited by TMHMM - 2.0 online tool. Their transmembrane domain, cytoplasmic domain (inside), and ER lumianl domain (inside) are shown.
(B) The amino acid sequences of RETR1 and RETR1-2 are aligned. Their TM regions and the putattive EBOV-GP recognition seqence are inidicatd. The the key cholesterol-binding site that determines oligomerization (E259 in RETR1, or E118 in RETR1-2) is shown in green and underlined.

bioRxiv preprint doi: https://doi.org/10.64898/2026.04.01.715898; this version posted April 2, 2026. The copyright holder for this preprint (which was not certified by peer review) is the author/funder, who has granted bioRxiv a license to display the preprint in perpetuity. It is made available under aCC-BY 4.0 International license.

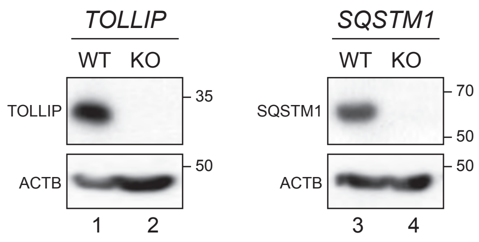

Figure S2. Generation of HEK293T TOLLIP-KO and SQSTM1-KO cells by CRISPR/Cas9.

bioRxiv preprint doi: https://doi.org/10.64898/2026.04.01.715898; this version posted April 2, 2026. The copyright holder for this preprint (which was not certified by peer review) is the author/funder, who has granted bioRxiv a license to display the preprint in perpetuity. It is made available under aCC-BY 4.0 International license.

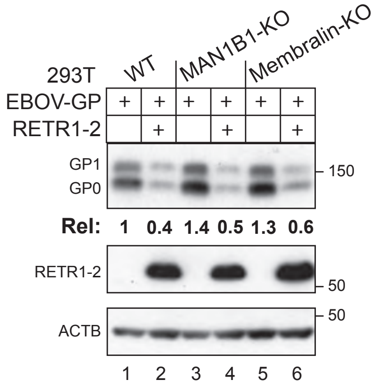

Figure S3. EBOV-GP and RETR1-2 were expressed in HEK293T and indicated KO cells and their expression was determined by WB.
